# Evaluation of Human Papilloma Virus (HPV) Genotyping and Viral Load Determination as Diagnostic Biomarkers of Cervical Cancer Risk

**DOI:** 10.3390/ijms24021320

**Published:** 2023-01-10

**Authors:** Marianna Martinelli, Chiara Giubbi, Laura Saderi, Rosario Musumeci, Federica Perdoni, Biagio Eugenio Leone, Robert Fruscio, Fabio Landoni, Andrea Piana, Giovanni Sotgiu, Clementina Elvezia Cocuzza

**Affiliations:** 1 Department of Medicine and Surgery, University of Milano-Bicocca, 20900 Monza, Italy; 2 Clinical Epidemiology and Medical Statistics Unit, Department of Medical, Surgical and Experimental Sciences, University of Sassari, 07100 Sassari, Italy; 3 Clinic of Obstetrics and Gynecology, San Gerardo Hospital, 20900 Monza, Italy; 4 Department of Medicine, Surgery and Pharmacy, University of Sassari, 07100 Sassari, Italy

**Keywords:** high-risk HPV, HPV, genotyping, viral load, HPV test

## Abstract

HPV testing in cervical cancer screening programs offers the possibility of introducing molecular standardized biomarkers for the triage of HPV-positive women. This study aimed to evaluate the role of HPV genotyping and viral load as possible diagnostic biomarkers of high-grade cervical lesions (CIN2+) by performing a preliminary evaluation of a new HPV test. Cervical specimens were obtained from 200 women referred for a colposcopy. Samples were tested using both Anyplex™ II HR-HPV as well as OncoPredict HPV^®^ Screening (SCR) and quantitative typing (QT). Using a cycle threshold cutoff (Ct) of 36.8 for the SCR assay and 1.27 log_10_ (viral copies/10^4^ cells) for the QT assay, relative clinical sensitivity for CIN2+ and relative clinical specificity for CIN2− as compared to Anyplex™ II HR-HPV were, respectively, 0.92 and 1.00 for SCR and 1.35 and 1.24 for QT. The distribution of high-risk HPV (HR-HPV) genotypes (*p* = 0.009) as well as the viral copy numbers (CIN2−: 3.7 log_10_ (viral copies/10^4^ human cells); CIN2+: 4.3 log_10_ (viral copies/10^4^ human cells); *p* = 0.047) were found to differ in women with high- and low-grade cervical lesions, suggesting a possible role of HPV genotyping and normalized viral load as potential biomarkers to identify women at increased risk of cervical lesions.

## 1. Introduction

Human papillomavirus (HPV) infection is considered a necessary cause for the onset of cervical cancer [1]. As a result, HPV DNA detection is now recommended as the primary screening method in cervical cancer prevention, as indicated by the WHO guidelines. Several molecular HPV assays based on different technologies have been developed and validated according to international criteria required for their introduction in screening programs [2].

Primary HPV-based cervical cancer screening offers the advantage of higher clinical sensitivity, associated with a higher negative predictive value, as compared to cytology, at the cost of a lower clinical specificity [3,4]. The reduced clinical specificity of HPV-based screening may, however, result in unnecessary colposcopy referrals. For this reason, HPV-positive women are presently triaged by Pap smear, and only those with confirmed abnormal cervical cytology undergo colposcopy [5,6].

The use of Pap smear or liquid-based cytology in the triage of HPV-positive women increases the specificity but has limitations: the performance is operator dependent, and the method is not easily reproducible and standardized. Moreover, cervical cytology cannot be performed on self-collected samples, which are increasingly being introduced in screening programs to improve the participation of women.

The introduction of more standardized and reproducible molecular methods for the triage of HPV-positive women, such as full high-risk HPV (HR-HPV) genotyping and genotype-specific HR-HPV viral load, could improve the risk stratification in cervical cancer screening programs.

Several studies have investigated the role played by HPV types and viral load in the development of low- and high-grade cervical lesions [7,8,9,10,11,12,13,14,15,16,17]. Full HR-HPV genotyping can guide the management of HPV-positive women [8,9,15] and allows the detection of coinfections that have been associated with the development of high-risk cervical lesions [16,17].

HPV genotype and associated viral load have also been described as important predictors of cervical intraepithelial neoplasia grade 2 or more (CIN2+) as well as cervical intraepithelial neoplasia grade 3 or more (CIN3+) [7]. Furthermore, serial HPV type-specific viral loads have been shown to predict the regression of CIN2 and CIN3 lesions [14].

The value of individual genotype viral loads has, however, been controversial. Dong and Colleagues demonstrated that the viral loads of HPV16, HPV31, HPV33, HPV52 and HPV58 are useful biomarkers of high-grade cervical lesions [13], whereas other studies reported that only HPV16 viral load is positively related to the severity of the cervical lesions [11,12]. More recently, another study demonstrated that the viral load of HPV16, HPV18 and HPV58 could be considered a diagnostic biomarker of high-grade cervical lesions [10]. Inconsistencies observed in these studies can be explained by the lack of standardized methods to assess viral load measurement. Previous reports are often based on relative light units for Hybrid Capture 2 (HC2) and cycle threshold (Ct) scores of PCR-based tests using different viral targets without any standard curve calibration or adjustment for the number of cells present in the sample as there are no clinically validated commercially available full HR-HPV genotyping assays allowing normalized viral load determination [10,11,12,13,14,18].

A preliminary evaluation of two new OncoPredict HPV^®^ multiplex real-time PCR assays (Hiantis, Italy) was performed, by comparing their clinical performance to that of Anyplex™ II HR-HPV (Seegene, Korea), a previously validated full HR-HPV genotyping assay. In particular, the two OncoPredict HPV^®^ assays, targeting E6/E7 genes, allow for a reflex testing algorithm, comprising a first-line qualitative screening (SCR), with partial genotyping for HPV16 and HPV18 with 11 other types detected as a pool (HPV 31, 33, 35, 39, 45, 51, 52, 56, 58, 59 and 68), followed by a second-line assay for the determination of normalized genotype-specific viral loads (QT) of 12 HR-HPV (HPV types 16, 18, 31, 33, 35, 39, 45, 51, 52, 56, 58 and 59).

## 2. Results

### 2.1. Study Population

Evaluation of the HPV molecular assays was carried out in a population of two hundred women referred for colposcopy with a recent history of cervical dysplasia. Biopsy was performed at the time of colposcopy in 152 out of the 200 referred women (76.0%), based on clinical judgment and local protocols. Overall, in the study population, 16 women (16/200; 8.0%) were shown to have SCC (squamous cervical cancer), whilst for 51 (51/200; 25.5%), 25 (25/200; 12.5%) and 17 (17/200; 8.5%) women a diagnosis of CIN3 (cervical intraepithelial neoplasia grade 3), CIN2 (cervical intraepithelial neoplasia grade 2) and CIN1 (cervical intraepithelial neoplasia grade 1) was made, respectively. In the remaining 43 women (43/200; 21.5%), no histological lesion was detected. Women with SCC, CIN3 and CIN2 lesions were classified as CIN2+ (92/200; 46%); all women with CIN1, with no histological lesions and/or with negative colposcopy (who did not undergo biopsy), were considered as CIN2− (108/200; 54%).

### 2.2. Cutoff Determination

**Figure 1:**
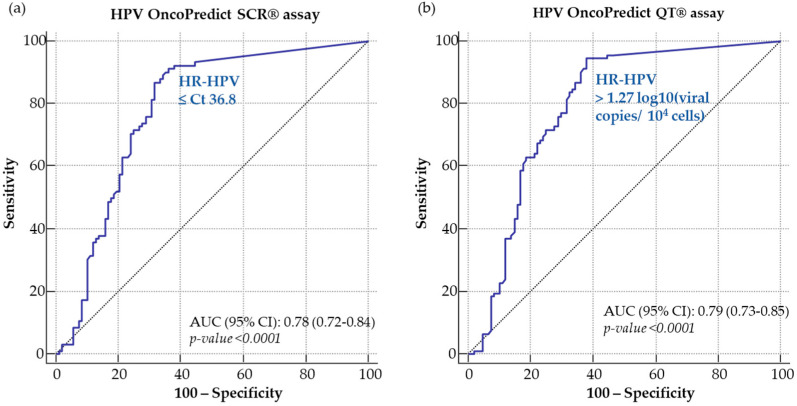
ROC curve analysis for the definition of cutoffs of HPV for OncoPredict HPV^®^ SCR (**a**) and QT (**b**) assays.

Based on the receiver operating characteristic (ROC) curve analysis, a preliminary positivity cutoff of Ct 36.8 was chosen for the SCR assay (Figure 1a) and 1.27 log_10_ (viral copies/10^4^ cells) was selected for the QT assay (Figure 1b).

**Figure 2:**
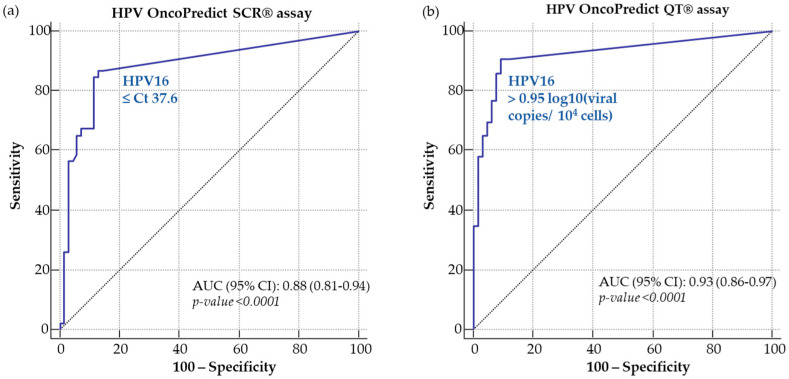
ROC curve analysis for the definition of cutoffs of HPV16 for OncoPredict HPV^®^ SCR (**a**) and QT (**b**) assays.

As HPV16 was the most prevalent genotype in the study population, a specific ROC curve analysis was performed to determine HPV16-specific cutoffs for both OncoPredict HPV^®^ assays: preliminary cutoffs corresponding to Ct 37.6 and 0.95 log_10_ (viral copies/10^4^ cells) were chosen for the SCR and QT assay, respectively (Figure 2a,b).

### 2.3. Analytical Agreement of OncoPredict HPV^®^ SCR and QT Assays Compared to Anyplex™ II HR-HPV

A substantial agreement in HR-HPV detection was found between OncoPredict HPV^®^ SCR and QT assays and Anyplex™ II HR-HPV (Seegene, Korea), (SCR: kappa = 0.77 (0.67–0.86); QT: kappa = 0.80 (0.71–0.89)). Anyplex™ II HR-HPV was previously clinically validated as part of the VALGENT-3 (VALidation of HPV GENotyping Tests) framework, an international cooperation designed for the comparison and clinical validation of HPV assays with genotyping capabilities [19]. A higher agreement of OncoPredict HPV^®^ assays with Anyplex™ II HR-HPV was shown for the detection of HPV 16 (SCR: kappa = 0.94 (0.89–0.99); QT: kappa = 0.88 (0.81–0.95)) (Table 1).

### 2.4. Clinical Performance of OncoPredict HPV^®^ SCR and QT Assays

The clinical sensitivity and specificity of OncoPredict HPV^®^ SCR and QT assays for the detection of CIN2+ were evaluated, and the clinical performance was compared to that of Anyplex™ II HR-HPV.

OncoPredict HPV^®^ SCR assay demonstrated a relative sensitivity of 0.92 and a relative specificity of 1.35, whilst OncoPredict HPV^®^ QT showed a relative sensitivity of 1.00 and a relative specificity of 1.24 (Table 2).

### 2.5. HR-HPV Prevalence and Genotype Distribution

Respectively, 141/200 (70.5%), 133/200 (66.5%) and 138/200 (69.0%) of women were found to be HR-HPV positive using Anyplex™ II HR-HPV, OncoPredict HPV^®^ SCR and OncoPredict HPV^®^ QT. HPV16 was detected in 56 women (28.0%) with Anyplex™ II HR-HPV, in 57 (28.5%) with OncoPredict HPV^®^ SCR and in 62 women (31.0%) with OncoPredict HPV^®^ QT. Clinical data and data on HPV positivity with OncoPredict HPV^®^ SCR and QT and Anyplex™ II HR-HPV are reported in Supplementary Materials.

A total of 79 women with a single HR-HPV type infection and 59 with coinfections with more than one of the 12 HR-HPV types, detected by the full genotyping OncoPredict HPV^®^ QT assay, were observed. In addition, 40/59 (67.8%), 12/59 (20.3%) and 7/59 (11.9%) women had multiple infections with, respectively, two, three and four HR-HPV genotypes. Table 3 shows the distribution of predominant HR-HPV in women with single and multiple HR-HPV infections.

No statistically significant differences were found in the distribution of coinfections among women with different grades of cervical dysplasia (CIN2+: 22/50; 44.0%; CIN2−: 37/88; 42.1%). The mean viral load in women with a single HR-HPV infection was lower than that in women with multiple infections, in whom the predominant genotype (genotype showing the highest viral load) was considered for comparison (single infection: 3.8 log_10_ (viral copies/10^4^ human cells); multiple infections: 4.1 log_10_ (viral copies/10^4^ human cells); *p*-value = 0.04).

Table 4 shows the different distribution of the predominant HPV genotypes detected by OncoPredict HPV^®^ QT in women with high-grade and low-grade cervical dysplasia.

### 2.6. HPV Viral Load

Among HPV-positive women, the mean viral load of the predominant genotype was higher in women with high-grade cervical dysplasia than in those with low-grade cervical dysplasia (CIN2+: 4.3 log_10_ (viral copies/10^4^ human cells); CIN2−: 3.7 log_10_ (viral copies/10^4^ human cells); *p*-value = 0.047).

## 3. Discussion

Infection with HR-HPV types is considered a necessary, but not sufficient, cause for the development of cervical cancer. HPV DNA tests offer improved clinical sensitivity, but lower clinical specificity, as compared to cervical cytology in predicting women at increased risk of developing cervical cancer. There is presently a great quest for new molecular biomarkers with increased specificity able to distinguish women with transient, clinically irrelevant HR-HPV infections from those at higher risk of progression to cervical cancer. Moreover, more specific molecular diagnostic biomarkers in the triage of HR-HPV-positive women could help to replace the use of subjective cervical cytology.

This study provides a preliminary evaluation of the clinical accuracy of two recently developed assays: OncoPredict HPV^®^ qualitative HR-HPV screening (SCR), with partial genotyping for HPV 16 and HPV 18, and a full genotyping quantitative assay (QT) for the normalized, type-specific viral load determination for 12 HR-HPVs.

In order to evaluate the performance of the novel OncoPredict HPV^®^ assays, clinical cutoffs were established based on ROC curve analysis following testing of cervical samples from women with documented high-grade lesions (CIN2+) and with low-grade lesions or negative colposcopy findings (CIN2−).

The OncoPredict HPV^®^ SCR and QT assays demonstrated overall good analytical concordance for HR-HPV and HPV16 detection in comparison to the previously validated Anyplex™ II HR-HPV assay. Small differences in positivity rates for HR-HPV infections observed with the three HPV assays could partially be explained by the fact that Anyplex™ II HR-HPV detects 14 different HR-HPV types, whilst OncoPredict HPV^®^ SCR and QT assays are able to identify 13 and 12 HR-HPV genotypes, respectively.

OncoPredict HPV^®^ SCR and QT assays were also shown to have a clinical sensitivity and specificity relative to Anyplex™ II HR-HPV similar to those reported for other assays validated for use in cervical cancer screening [20,21]. The preliminary analysis identified clinical cutoffs for HPV16 that were different from those set up for grouped HR-HPV types, suggesting that, for each HR-HPV genotype, different cutoffs may need to be defined through larger population studies [17,22]. Due to the limited sample size of enrolled women, in this preliminary study, it was not possible to evaluate individual viral load cutoffs for other HR-HPV types. Moreover, future validation studies of OncoPredict HPV^®^ SCR and QT assays will need to be performed on a larger number of samples, as indicated by the international criteria for the validation of HR-HPV tests [2,23].

The statistically significant different distribution of HR-HPV types in women with high- and low-grade cervical lesions further supports the importance of full genotyping HPV assays for the triage of HR-HPV-positive women. As previously reported [7,8,24], HPV16 and HPV33 were most frequently associated with CIN2+ lesions. Interestingly, in agreement with the findings of Cuzick and colleagues [8], HPV18, which is often associated with cervical cancer [25], was not frequently detected in this preliminary study among women with high-grade cervical lesions.

Previous studies have shown an association between multiple HR-HPV infections and high-grade cervical lesions [16,17], whilst others have not confirmed this finding [7,26]. The present study did not demonstrate a different prevalence of coinfections among the two groups of women with CIN2+ and CIN2− lesions. Interestingly, viral loads were higher in women with coinfections.

Several studies are reporting that viral load may be a candidate biomarker for the triage of HPV-positive women [10,17,27], particularly in the case of HPV16-associated infections [28,29]. In this study, women with CIN2+ lesions were shown to have significantly higher mean viral loads than those with CIN2− lesions, by means of the OncoPredict HPV^®^ QT assay. Furthermore, accurate quantification of sample cellularity allows the determination of a normalized, genotype-specific viral load, as recently advocated by the European Society of Gynaecologic Oncology (ESGO) and the European Federation of Colposcopy [18]. Moreover, the quantification of the number of cells, by means of a human gene present in a constant copy number per cell (CCR5 in OncoPredict HPV^®^ QT assay), allows assessment of the sample adequacy, an important quality control in HPV screening programs particularly if based on self-sampling. Thanks to a correct evaluation of cellularity, it is possible to identify a sample with a low number of cells that should be considered invalid and whose collection should be repeated.

The main limitations of this preliminary study are the relatively small number of investigated women as well as their enrolment in a colposcopy rather than in a cervical cancer screening setting. As a result, the prevalences of HPV-positive women, multiple infections and viral loads might not reflect those of a general screening population.

Finally, the clinical validation of OncoPredict HPV^®^ SCR and QT assays, according to international criteria [2], will require a well-defined set of clinical samples, from screening and diseased populations, such as those described by the VALGENT Protocol [23]. The VALGENT consortium provides representative samples from screening and diseased populations to allow the complete validation of new HPV kits by assessment of non-inferior sensitivity and specificity to a standard comparator test and intra- and inter-laboratory reproducibility.

## 4. Materials and Methods

### 4.1. Study Population and Sample Collection

From November 2017 to July 2021, 200 women referred for colposcopy with a recent history of cervical dysplasia were enrolled as part of an ongoing study approved by the Ethical Committee of the University of Milano-Bicocca, Monza, Italy (Protocol no. 0037320/2017 and 0086409/2018). The study was conducted in accordance with the Declaration of Helsinki, and informed consent was obtained from all subjects involved in the study.

During the gynecological examination, women underwent biopsy and/or conization based on the local clinical protocols and on the outcome of the colposcopy. The classification of histological outcomes was conducted according to the WHO’s histological classification of tumors (2020) [30].

A cervical sample was collected by the clinician, from all women undergoing colposcopy, which was immediately suspended in 20 mL of ThinPrep^®^ PreservCyt^®^ (Hologic, Marlborough, MA, USA). Cervical samples were then transported to the Laboratory of Clinical Microbiology of the Department of Medicine and Surgery, University of Milano-Bicocca, Italy, where they were processed.

### 4.2. Nucleic Acid Extraction, HPV Typing and Viral Load Quantification

Cervical specimens were vigorously vortexed for 30 s, aliquoted and stored at −20 °C until nucleic acid extraction.

Nucleic acids were extracted with a StarMag 96 × 4 Universal Cartridge (Seegene, Seoul, Korea), starting with 200 μL of sample, using the automated workstation Microlab Nimbus. Nucleic acids were then eluted in 100 μL and tested using the Anyplex™ II HR-HPV real-time PCR assay (Seegene, Seoul, Korea), according to the manufacturer’s instructions.

A separate 400 uL aliquot of the same sample was processed on the automated Fluent 480 liquid handler (Tecan, Switzerland), allowing nucleic acid extraction to be performed using *Quick*-DNA/RNA MagBead (Zymo, Irvine, CA, USA) and subsequently real-time PCR plate preparation using OncoPredict HPV^®^ assays (Hiantis, Milan, Italy), according to the manufacturer’s instructions.

The Anyplex™ II HR-HPV assay potentially detects 14 HR-HPVs (16, 18, 31, 33, 35, 39, 45, 51, 52, 56, 58, 59, 66 and 68) using type-specific melting profiles. The OncoPredict HPV^®^ solution is based on 2 separate assays, SCR and QT, which can be used independently or as reflex testing. The SCR assay is a qualitative assay allowing the separate detection of HPV16, HPV18 and one or more of 11 “other” HR-HPVs (31, 33, 35, 39, 45, 51, 52, 56, 58, 59, 68); the QT assay detects and quantifies 12 HR-HPVs (16, 18, 31, 33, 35, 39, 45, 51, 52, 56, 58 and 59) and uses *C-C chemokine receptor type 5* (CCR5) to evaluate sample adequacy and cellularity. HPV individual viral loads are expressed as log_10_ (normalized viral copies/10^4^ human cells).

The three real-time PCR assays were performed on a CFX96 PCR system (Bio-Rad, Hercules, CA, USA) using 5 μL of template DNA in a total reaction volume of 20 μL for Anyplex™ II HR-HPV and in a total volume of 15 μL for both OncoPredict HPV^®^ assays.

### 4.3. Statistical Analysis

Descriptive statistics were used to summarize data. Comparison between quantitative variables was performed using Student’s *t* test, whereas the chi square test was used for qualitative ones.

Receiver operating characteristic (ROC) curves were used to assess the performances of both OncoPredict HPV^®^ assays for the detection of high-grade cervical lesions at different positivity thresholds. For the OncoPredict HPV^®^ QT assay, the analysis was performed on log_10_-transformed data. Cutoff points were chosen on the basis of the best clinical sensitivity and specificity (i.e., assessment of diagnostic accuracy).

Based on the cutoffs determined by the ROC curve analysis, the performance of the OncoPredict HPV^®^ SCR and QT assays was evaluated by comparing HR-HPV results obtained with each assay in comparison to Anyplex™ II HR-HPV, previously clinically validated as part of the VALGENT-3 framework [19].

For the OncoPredict HPV^®^ QT assay, in case of coinfections, the HPV type with the highest viral load was used in the evaluation, for OncoPredict HPV^®^ SCR, the HPV type with higher viral loads (lower Ct value) was used.

Analytical agreement in HR-HPV detection obtained with different tests was evaluated with the Cohen’s kappa (κ) statistics. Agreement was defined as slight (0.00 < k < 0.20), fair (0.20 < k < 0.40), moderate (0.41 < k < 0.60), substantial (0.61 < k < 0.80) and almost perfect (0.81 < k < 1.00). A two-tailed *p*-value less than 0.05 was considered statistically significant. The statistical software STATA version 17 (StataCorp, College Station, TX, USA) and MedCalc Statistical Software version 20.015 (MedCalc Software bvba, Ostend, Belgium; 2019) were used to perform statistical computations.

## 5. Conclusions

Preliminary evaluation on the clinical performance of OncoPredict HPV^®^ SCR and QT assays provided insights on the use of HPV genotyping and normalized viral load as potential diagnostic biomarkers to identify women at increased risk of cervical lesion.

The full validation of the HPV assays on a larger cohort of women coming from a screening setting will confirm the role of these molecular biomarkers in cervical cancer prevention programs. Further studies evaluating the use HPV genotyping and normalized viral load in the follow up of HPV-positive women might also provide information on their use in the management and follow up of women at increased risk of disease progression.

## Figures and Tables

**Table 1:** Analytical agreement in HR-HPV and HPV16 detection between OncoPredict HPV^®^ SCR and QT assays and Anyplex™ II HR-HPV.

	Agreement between OncoPredict HPV^®^ SCR and Anyplex^TM^ II HR-HPV		Agreement between OncoPredict HPV^®^ QT and Anyplex^TM^ II HR-HPV	
	HPV16	HR-HPV	HPV16	HR-HPV
Positive Agreement n (%)	54/56 (96.4)	127/141 (90.1)	54/56 (96.4)	131/141 (92.9)
Negative Agreement n (%)	141/144 (97.9)	53/59 (89.8)	136/144 (94.4)	52/59 (88.1)
Overall Agreement n (%)	195/200 (97.5)	180/200 (90.0)	190/200 (95.0)	182/200 (91.0)
Kappa (95% CI)	0.94 (0.89–0.99)	0.77 (0.67–0.86)	0.88 (0.81–0.95)	0.80 (0.71–0.89)

**Table 2:** Absolute and relative clinical sensitivity and specificity for high-grade cervical lesions.

Real-Time Assay	Clinical Sensitivity (%) (95% CI)	Clinical Specificity (%) (95% CI)	Relative Sensitivity to Anyplex™ II HR-HPV (95% CI)	Relative Specificity to Anyplex™ II HR-HPV (95% CI)
Anyplex™ II HR-HPV	94.6 (87.9–97.6)	50.0 (40.7–59.2)	-	-
OncoPredict HPV^®^ SCR	87.0 (78.3–93.1)	67.6 (57.9–76.3)	0.92 (0.84–1.01)	1.35 (1.08–1.70)
OncoPredict HPV^®^ QT	94.6 (87.8–98.2)	62.0 (52.2–71.2)	1.00 (0.93–1.07)	1.24 (0.98–1.58)

**Table 3:** HR-HPV genotype distribution in women with single and multiple HR-HPV infections.

Predominant Genotype	Single Infection (n = 79)	Coinfections (n = 59)	*p*-Value
HPV16 n (%)	33 (41.8)	14 (23.7)	0.06
HPV18 n (%)	3 (3.8)	6 (10.2)	
HPV31 n (%)	18 (22.8)	10 (17.0)	
HPV33 n (%)	1 (1.3)	3 (5.1)	
HPV35 n (%)	2 (2.5)	1 (1.7)	
HPV39 n (%)	2 (2.5)	3 (5.1)	
HPV45 n (%)	4 (5.1)	3 (5.1)	
HPV51 n (%)	3 (3.8)	7 (11.9)	
HPV52 n (%)	2 (2.5)	3 (5.1)	
HPV56 n (%)	3 (3.8)	0 (0.0)	
HPV58 n (%)	7 (8.9)	4 (6.8)	
HPV59 n (%)	1 (1.3)	5 (8.5)	

**Table 4:** HR-HPV genotype distribution detected in women with high-grade and low-grade cervical dysplasia by OncoPredict HPV^®^ QT assay.

Predominant Genotype	CIN2− HPV Positive (n = 50)	CIN2+ HPV Positive (n = 88)	*p*-Value
HPV16 n (%)	8 (16.0)	39 (44.3)	0.009
HPV18 n (%)	4 (8.0)	5 (5.7)	
HPV31 n (%)	9 (18.0)	19 (21.6)	
HPV33 n (%)	1 (2.0)	3 (3.4)	
HPV35 n (%)	2 (4.0)	1 (1.1)	
HPV39 n (%)	4 (8.0)	1 (1.1)	
HPV45 n (%)	2 (4.0)	5 (5.7)	
HPV51 n (%)	7 (14.0)	3 (3.4)	
HPV52 n (%)	2 (4.0)	3 (3.4)	
HPV56 n (%)	2 (4.0)	1 (1.4)	
HPV58 n (%)	6 (12.0)	5 (5.7)	
HPV59 n (%)	3 (6.0)	3 (3.4)	

## Supplementary Materials

The following supporting information can be downloaded at: https://www.mdpi.com/article/10.3390/ijms24021320/s1.
